# Aerobic biogenesis of selenium nanospheres by *Bacillus cereus *isolated from coalmine soil

**DOI:** 10.1186/1475-2859-9-52

**Published:** 2010-07-05

**Authors:** Soniya Dhanjal, Swaranjit Singh Cameotra

**Affiliations:** 1 Institute of Microbial Technology, Sector 39-A, Chandigarh 160036, India

## Abstract

**Background:**

Microorganisms that are exposed to pollutants in the environment, such as metals/metalloids, have a remarkable ability to fight the metal stress by various mechanisms. These metal-microbe interactions have already found an important role in biotechnological applications. It is only recently that microorganisms have been explored as potential biofactories for synthesis of metal/metalloid nanoparticles. Biosynthesis of selenium (Se^0^) nanospheres in aerobic conditions by a bacterial strain isolated from the coalmine soil is reported in the present study.

**Results:**

The strain CM100B, identified as *Bacillus cereus *by morphological, biochemical and 16S rRNA gene sequencing [GenBank:GU551935.1] was studied for its ability to generate selenium nanoparticles (SNs) by transformation of toxic selenite (SeO_3_^2-^) anions into red elemental selenium (Se^0^) under aerobic conditions. Also, the ability of the strain to tolerate high levels of toxic selenite ions was studied by challenging the microbe with different concentrations of sodium selenite (0.5 mM-10 mM). ESEM, AFM and SEM studies revealed the spherical Se^0 ^nanospheres adhering to bacterial biomass as well as present as free particles. The TEM microscopy showed the accumulation of spherical nanostructures as intracellular and extracellular deposits. The deposits were identified as element selenium by EDX analysis. This is also indicated by the red coloration of the culture broth that starts within 2-3 h of exposure to selenite oxyions. Selenium nanoparticles (SNs) were further characterized by UV-Visible spectroscopy, TEM and zeta potential measurement. The size of nanospheres was in the range of 150-200 nm with high negative charge of -46.86 mV.

**Conclusions:**

This bacterial isolate has the potential to be used as a bionanofactory for the synthesis of stable, nearly monodisperse Se^0 ^nanoparticles as well as for detoxification of the toxic selenite anions in the environment. A hypothetical mechanism for the biogenesis of selenium nanoparticles (SNs) involving membrane associated reductase enzyme(s) that reduces selenite (SeO_3_^2-^) to Se^0 ^through electron shuttle enzymatic metal reduction process has been proposed.

## Background

Selenium (Se), belonging to group 16 of the periodic table is well known for its photoelectric and semiconductor properties. It is used in solar cells, rectifiers, photographic exposure meters and xerography [1]. Amorphous selenium nanoparticles (SNs) possess unique photoelectric, semiconducting and X-ray-sensing properties. These nanoparticles also show biological activity and good adsorptive ability due to interaction between the nanoparticles and NH, C = O, COO^_ ^and C-N groups of proteins [2]. Selenium nanoparticles have also been developed for applications in medical diagnostics [3]. Studies on the biological toxicity of selenium and its nanoforms revealed that nano-selenium showed equal efficiency in increasing the activities of glutathione peroxidase and thioredoxin reductase [4]. Gao et al. [5] demonstrated the antioxidant properties of hollow spherical nanoparticles of selenium. Similar observations that nano-Se can serve as an antioxidant with reduced risk of selenium toxicity was reported by Wang et al. [6]. The size of nanoparticles play an important role in their biological activity as 5-200 nm nano-Se can directly scavenge free radicals *in vitro *in a size-dependent fashion [7]. Several methods including γ-irradiation and laser ablation have been applied to synthesize selenium nanoparticles but most widely used synthetic approach for preparing selenium nanoparticles is chemical reduction [8].

Recently, there has been increasing interest in synthesis of nanoparticles using biological systems leading to the development of various biomimetic approaches. Microorganisms, such as bacteria, yeast and fungi play an important role in recycling of minerals in the environment. Some of these microorganisms can survive and grow even at high metal ion concentrations. The toxicity of metal ions is reduced or eliminated by changing the redox state of the metal ions and in the process leading to the formation of well-defined nanoscale particles in some cases [9].

Selenium occurs in variety of oxidation states like selenate (SeO_4_^2-^)/selenite (SeO_3_^2-^) oxyions, wherein the oxidation states are + 6 and + 4; elemental selenium (Se^0^) and selenide (Se^2-^). The toxicity of these states is related to their degrees of solubility in water and hence their bioavailability. Elemental selenium can exist in forms other than red amorphous selenium (Se^0^) as selenate (SeO_4_^2-^)/selenite (SeO_3_^2-^) which are highly water soluble and as selenide (Se^2-^) which is gaseous in nature. Among the various selenium species, selenite (SeO_3_^2-^) reduction has attracted a great deal of attention as potential compound for microbial reduction due to its high toxicity. Se-reducing bacteria are ubiquitous and occur in diverse terrestrial and aquatic environments [10]. A few microorganisms have been well characterized for their ability to reduce toxic selenate and selenite oxyions into non-toxic elemental form Se^0 ^under aerobic and anaerobic conditions [10-13].

The biogenesis of selenium nanostructures during the dissimilatory respiration was reported by Oremland et al. [14] during the dissimilatory respiration. Se^0 ^particles formed by the Se-respiring bacteria *Sulfurospirillum barnesii*, *Bacillus selenitireducens *and *Selenihalanaerobacter shriftii *are structurally unique compared to elemental selenium formed by chemical synthesis. The three anaerobes used toxic selenium oxyions as the electron acceptors during anaerobic respiration which resulted in the formation of stable, uniform nanospheres of selenium (diameter ~ 300 nm). The majority of studies on the biogenesis of selenium nanoparticles have focused on anaerobic systems. However, anaerobic conditions have limitations, such as culture conditions and isolate characteristics that make optimization and scale-up in bio-manufacturing processes tedious and challenging [15]. Selenium-tolerant aerobic microorganisms may provide an opportunity to overcome these limitations in the biosynthetic processes. Very few studies have reported the aerobic formation of selenium nanoparticles by microorganisms. The generation of selenium nanospheres by soil bacteria *Pseudomonas aeruginosa and Bacillus *sp. under aerobic conditions has recently been reported [15,16]. These studies include the partial characterization of selenium nanospheres formed by the two isolates. The aim of the present investigation was to study the possible formation of selenium (Se^0^) nanospheres in aerobic conditions by a Se-reducing bacterial strain (CM100B) isolated from a coalmine soil of West Bengal, India.

## Methods

### Microorganism and Growth Conditions

The strain CM100B was isolated from coalmine soil (coal mines located in Asansol, Latitude: 23°41' N and Longitude: 86°59'E, West Bengal, India) by enrichment of the soil sample for one week with sodium selenite (0.5 mM) followed by standard method of dilution plating on tryptic soy agar (TSA) medium supplemented with 0.5 mM sodium selenite. The pure isolate was routinely cultured on TSA plates containing 2 mM selenite at 37°C.

### Morphological, Biochemical and Physiological characterization of the isolate CM100B

Biochemical characterization of the strain CM100B was performed following standard methods as described in Bergy's Manual of Systemic Bacteriology, Vol. 1, and Manual for the Identification of Medical Bacteria by Cowan & Steel (Second edition, Cambridge University press).

### 16s rRNA gene sequencing and phylogenetic analysis

For the 16S rRNA gene analysis the genomic DNA was extracted by the CTAB method followed by PCR amplification with universal primers 27F and 1492R. Sequencing of the amplified product was done by dideoxy chain terminator method using the Big Dye terminator kit followed by capillary electrophoresis on an ABI 310 genetic analyzer (Applied Biosystems, USA). The sequence obtained was BLAST searched and compared with sequences of other closely related species retrieved from the GenBank database http://www.ncbi.nlm.nih.gov/BLAST/ followed by alignment using the MEGA software version 4 [17]. A phylogenetic tree was constructed using the neighbor-joining algorithm. Bootstrap analysis was performed to assess the confidence limits of the branching.

### Bacterial growth under selenite stress

The effect of selenite on the growth of the bacterial isolate was determined in the presence of 0.5 mM, 1 mM, 2 mM, 5 mM and 10 mM of sodium selenite. Sodium selenite was prepared as 1 M stock solution and sterilized by filtration. 250 ml Erlenmeyer flasks containing 100 ml of Tryptic Soya Broth (TSB) supplemented with respective concentrations of selenite were inoculated with overnight grown bacterial culture and incubated at 37°C at 200 rpm. Bacterial growth was measured by the quantification of total protein content of microbial biomass. Protein concentration in bacterial cell extracts was determined by using the Bicinchoninic acid (BCA) method with Bovine Serum Albumin (BSA) as standard. A 1 ml aliquot of bacterial culture was collected at different time intervals of bacterial growth and was centrifuged at 4722 × *g *(Sigma 1-14) for 10 min. The pellet was resuspended in 100 μl of extraction buffer (50 mM Na_2_HPO_4_, pH 7; 10 mM β-mercaptoethanol; 10 mM Na_2_- EDTA; 0.1% Sodium dodecyl sulphate (SDS); 0.1% Triton X-100). The resulting suspension was sonicated for 5 min and centrifuged at 10625 × *g *for 15 min at 4°C. The supernatant was collected and measured for protein content. Flask with inoculum without the addition of selenite served as control.

### Reduction of selenite by strain CM100B

To determine the reduction of selenite by the bacterial isolate, the organism was exposed to 2 mM selenite. Samples were collected at 2 h intervals and centrifuged at 1844 × *g *to separate the bacterial biomass and the supernatant. Se content in the supernatants was determined by Atomic absorption spectrophotometer (AA-6800, Shimadzu) in Hydride Vapor Generation mode, with a selenium cathode lamp. An air-acetylene (oxidizing) flame was used and a wavelength of 196 nm was chosen for the purpose of absorption of incident light.

### Flow Cytometry

The relative decrease in the size of the cell population under selenite stress was determined by Forward Scatter using FACS Calibur (Becton-Dickinson). The strain CM100B was grown in the presence of 5 mM sodium selenite. An aliquot of 1 ml bacterial culture was collected at regular time intervals of bacterial growth to determine the cell size. The samples were centrifuged at 1180 × *g *for 10 min. The cell pellet was gently washed twice with phosphate buffered saline (PBS) pH 7.2 and re-suspended in it for analysis. Culture without addition of selenite oxyions served as control.

### Microscopic studies

#### Environmental Scanning Electron Microscopy (ESEM)

ESEM examination of the culture was done by growing the cells in the presence of 2 mM selenite for 24 h. The sample was applied directly on the stub and scanned with a Hitachi Scanning Electron Microscope under variable pressure in ESEM mode.

#### Scanning Electron Microscopy

Strain CM100B was grown in TSB supplemented with 2 mM sodium selenite at 37°C. After 24 h of incubation, cells were centrifuged at 1844 × *g *at 4°C for 10 min and scanning electron microscopic studies were performed on the processed samples. Sample processing involves washing, fixing and drying of cells. Harvested cells were washed thrice with phosphate buffer saline (PBS, pH 7.4) and layered onto polylysine coated cover slips. Fixation was done with modified Karnovsky's fixative (2% paraformaldehyde and 2.5% glutaraldehyde in 0.1 M sodium phosphate buffer, pH 7.4). Cells were again washed with PBS and distilled water. Fixed cells were dehydrated through a series of alcohol dehydration steps (30%, 50%, 70%, 90% and 100%) and finally layered with t-butyl alcohol for freeze drying and sputter coated. The samples were then viewed under Scanning Electron Microscope (Carl Zeiss NTS, GmbH, Germany).

#### Atomic Force Microscopy

The samples were centrifuged at 4722 × *g *for 10 min. The acquired cell pellet was gently washed twice with deionized-distilled water and re-suspended in it. 5 μl cell suspension was put on the freshly cleaved mica surface and then immediately dried with nitrogen gas. All AFM experiments were carried out by NTMDT Solver pro 7 Atomic Force Microscope using a Si tip. AFM imaging was done in tapping mode.

#### Transmission Electron Microscopy (TEM)

For ultrastructural studies, 24 h old culture grown in the presence of 2 mM sodium selenite was centrifuged at 1844 × *g *for 15 min, washed thrice with 10 mM phosphate buffer, pH 7.4 and fixed for 10-12 h at 4°C in modified karnovsky's fixative. After successive washings in 10 mM sodium phosphate buffer pH 7.4 cells were post fixed in 1% osmium tetroxide in the same buffer. After several washes in the same buffer, cells were dehydrated in graded acetone solutions (30%, 50%, 70%, 90% and 100% for 15 min each) and embedded in CY 212 araldite (10 ml), along with Dodecenyl Succinic Anhydride (DDSA) as hardner (10 ml), and tri(dimethylaminomethyl) phenol (DMP-30) as catalyst (0.4 ml). Ultrathin sections of 60-80 nm thickness were cut using an ultracut E (Reichert Jung) ultra-microtome and the sections stained with alcoholic uranyl acetate (saturated solution in ethanol) for 2 min and subsequently in lead citrate for 2 min before examining the grids in HRTEM Technai G20-stwin (200 kV) Transmission Electron Microscope. Cells grown for the same time period without the addition of the selenite were taken as control and processed similarly.

#### Elemental composition analysis with energy dispersive X-ray (EDX)

To ascertain the reduction of Se^4 + ^to elemental selenium (Se^0^) the samples were processed by a method similar to that used for TEM studies. The selected areas within TEM sections were subjected to elemental composition analysis using an EDX (Bruker AXS Inc. USA, Quantax-200) micro-analysis system coupled to a Transmission Electron Microscope. Sample collected from the culture without addition of selenite (SeO_3_^2-^) was taken as control.

#### Recovery of selenium nanoparticles from the culture broth

Bacterial strain inoculated in 300 ml TSB amended with 2 mM sodium selenite and incubated at 37°C at 200 rpm for 48 h. The culture broth was centrifuged at 10020 × *g *(Hermle centrifuge, Z36HK) at 4°C for 10 min. The pellet was discarded and the cell-free medium was centrifuged at 41410 × *g *at 4°C for 30 min. The supernatant was discarded and the pellet with the selenium-containing particles was re-suspended in water. The suspension was washed twice by repeating the two centrifugation steps.

#### Characterization of reduced selenite

The absorption spectra of the red elemental selenium particles suspended in aqueous solution was recorded using a Hitachi U2800 spectrophotometer by wavelength scan from 300-1100 nm setting the baseline with water. TEM studies were carried out using JEM 2100 (JEOL) microscope operating at 120 kV accelerating voltage. Samples were prepared by placing a drop of selenium particles suspended in water on carbon-coated TEM grids. The film on the TEM grids was allowed to dry for 5 min at room temperature before analysis. Charge distribution (zeta potential) was analyzed using dynamic light scattering system (Beckman Coulter, USA) by illuminating the solution of selenium particles with He-Ne Laser (633 nm) in a sample cell.

#### Determining the membrane-associated reductase activity

The culture was grown to log phase and centrifuged at 3910 × *g *(Hermle centrifuge, Z36HK) for 10 min at 4°C to obtain the cell pellet. Pellet was washed with 10 mM Tris-Cl (pH 7.5) twice and re-suspended in the same buffer for sonication. After sonication, the cell lysate was centrifuged at 22540 × *g *for 40 min to separate the soluble and membrane fractions. The total protein content was estimated by Bradford method using BSA as standard. Selenite reductase activity was determined using the following reaction mixture: 5 ml TSB, 100 μg of protein, 5 mM sodium selenite. The reaction mixture was incubated at 37°C for 3-4 h. Reaction mixture without addition of membrane or soluble fractions served as controls.

## Results and Discussion

### Characterization of selenite tolerant bacterium isolated from coalmine soil

**Figure 1:**
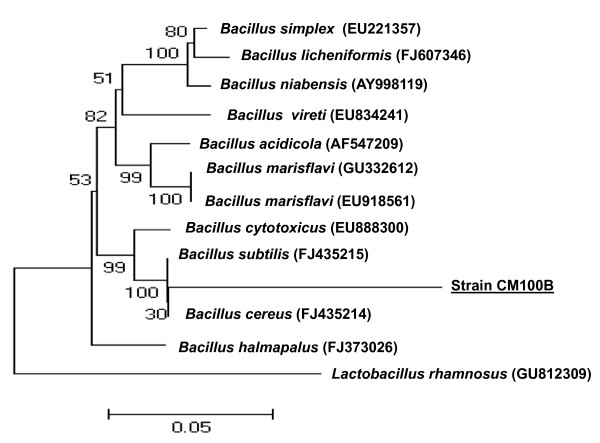
**The Neighbor-Joining (NJ) tree, inferred using MEGA software version 4 (Tamura et al. 2007)**. Bootstrap values expressed as percentage of 1000 replications are given at the nodes. Bar equals 0.05% sequence variation.

Selenium is a very coalphile element, having strong affinity to organic and inorganic matter present in coals. The average selenium content in hard coals and brown coals is estimated to be 1.6 ± 0.1 and 1.0 ± 0.15 ppm, respectively [18]. Therefore, there was high probability of finding an efficient selenium transforming microbe from the collected coalmine soil. The enrichment of coalmine soil with selenite oxyions for over a period of 7 days resulted in isolation of selenite tolerant bacterial strain CM100B. The bacterial isolate was subjected to characterization by morphological, biochemical and molecular methods. It is a Gram-positive, facultative anaerobic, spore-forming, rod shaped, flagellated bacterium. Temperature for growth ranged from 15°C-42°C (optimum 37°C), pH values 5-11 (optimum pH 7.5), salt concentration (NaCl %) up to 7.5%. Biochemical analysis shows that it is positive for catalase, oxidase, hydrolysis of gelatin, starch, casein and negative for citrate, methyl red, Voges-Proskauer, nitrate, urea hydrolysis and oxidation/fermentation tests. Acid is produced from carbohydrates namely dextrose, fructose, maltose, mannose, sucrose and trehalose. The results of 16S rRNA (1439 bp) gene sequence analysis of the strain suggested that it belongs to the genus *Bacillus *sp. [GenBank:GU551935.1]. In the phylogenetic tree the strain CM100B formed a coherent branch with *Bacillus cereus *(Fig. 1)The isolate CM100B also showed high sequence similarity of 99% to *Bacillus cereus*. Therefore, on the basis of the above results the strain CM100B was characterized as *Bacillus cereus*.

### Growth on selenite (SeO_3_^2-^) oxyion and evaluation of selenite (SeO_3_^2-^) reducing ability

**Figure 2:**
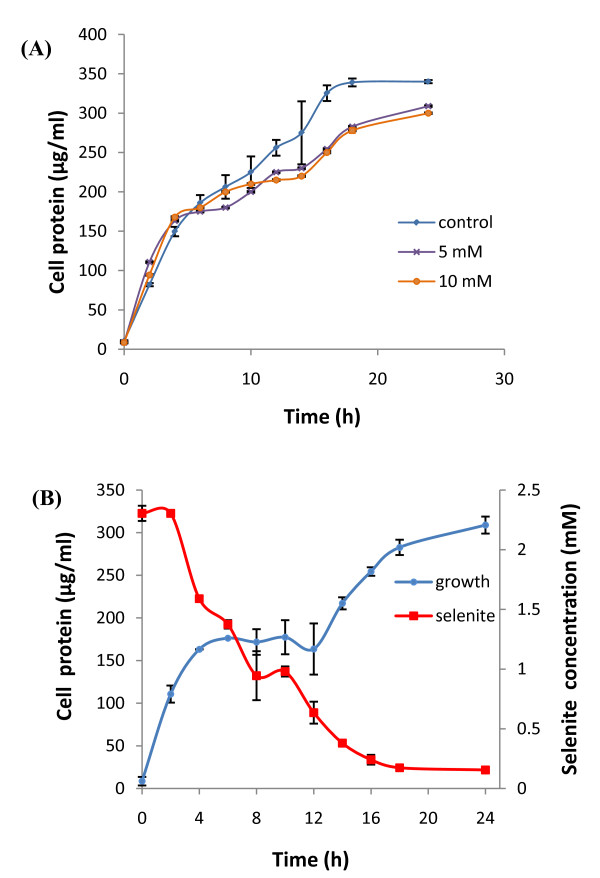
**Growth of strain CM100B under selenite stress and selenite reduction**. **(A) **Growth profile of strain CM100B in the presence of high concentrations of sodium selenite. **(B) **Time course of microbial growth and selenite (SeO_3_^2- ^) reduction by the strain CM100B.

**Figure 3:**
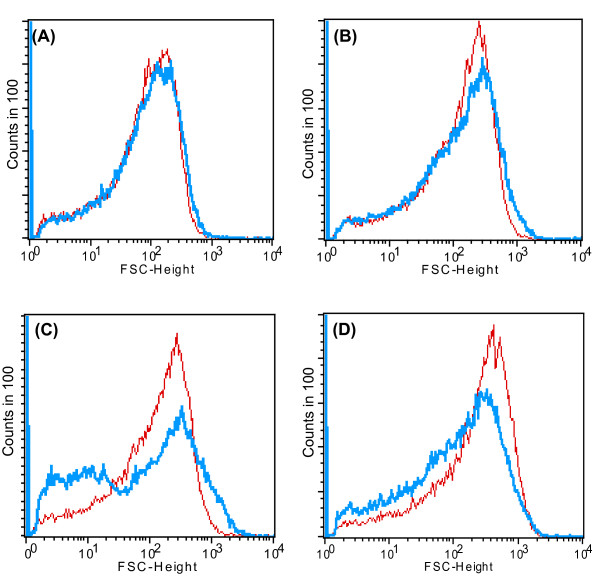
**Gradual decrease in average bacterial cell size which was grown in the presence of selenite oxyions as measured by flow cytometry**. X axis is log scale and Y axis indicates the number of bacterial cells (counts in 100). Shift towards left/right on the X axis indicates decrease/increase in cell size in Forward Scattering analysis. Test population (blue) and control population (red). **(A) **After 12 h of bacterial growth. **(B) **After 24 h of bacterial growth. **(C) **After 36 h of bacterial growth and **(D) **After 48 h, the test population shifts towards left indicating the decrease in cell size of test population which was exposed to selenite stress.

In order to determine the toxicity of selenite (SeO_3_^2-^) to the microorganism, the growth profile of the bacteria was studied by addition of different concentrations of sodium selenite (0.5 mM, 1 mM, 2 mM, 5 mM and 10 mM) in the growth medium under aerobic conditions. The strain CM100B formed reddish cell suspension which indicated its ability to reduce the toxic, colorless, soluble selenite (SeO_3_^2-^) ions to nontoxic, red elemental insoluble form of selenium (Se^0^). It is worth noting that the formation of red precipitate of elemental selenium started after 2 h of exposure to selenite oxyions which made it difficult to study the growth profile of the strain under selenite stress by spectroscopy due to optical interference from Se^0 ^particles in spectrophotometric measurements. Therefore, the total protein content was estimated at regular time intervals and correlated with the increase in the growth of the microorganism. The growth profile of the strain in the presence of selenite (0.5mM-10mM) was comparable to that of control without addition of selenite (Fig. 2A, Graphically growth is shown in the presence of 5 mM and 10 mM selenite for clarity, however growth studies were done in the presence of selenite concentrations ranging from 0.5-10 mM as mentioned above). Simultaneous protein estimation by BCA method indicated that very short lag period occurred during the growth of the microbe either in the presence or absence of selenite. Apparently, selenite was observed to be non-toxic to the strain at the above mentioned concentrations. As evident from the Fig. 2B, there was rapid decrease of selenite oxyions in the culture broth within a period of 24 h. Viable cultures challenged with toxic selenite oxyions begin to turn red within 2-3 h of bacterial growth. A marginal decrease in selenite (SeO_3_^2-^) concentrations as a result of abiotic reduction by the TSB media was also observed in the uninoculated flasks (data not shown). There was a gradual decrease in average bacterial cell size which was grown in the presence of selenite oxyions as compared to the control cells which were grown without the addition of selenite oxyions. After 12 h and 24 h of bacterial growth, the size of the test and the control population was equivalent (Fig. 3A and 3B). A gradual decrease in cell size was observed after 36 h of cell growth in the presence of selenite oxyions (Fig. 3C). At 48 h of bacterial growth the test cell population under selenite stress was comparatively smaller in size than the control cells which were grown without the addition of selenite as determined by Forward Scatter in flow cytometric analysis (Fig. 3D)Under the stressful condition of toxic selenite ions the morphology of the cells is altered resulting in decrease in cell size. Possibly, the changes in cell morphology could be explained by the surface/volume ratio. The organisms reduce their cell size and increase their relative surface area for better uptake of the nutrients for survival under environmental stress conditions. The purpose of the growth profile studies was to understand the response of the test organism to high concentrations of selenite which are several orders of magnitude higher than usual levels present in the environment [19].

The above results indicate that reduction of selenite (SeO_3_^2-^) by the microbe occurred more rapidly than was observed in earlier reports [11,16]. Dungan et al. [19] reported formation of Se^0 ^after 28 h during studies with *Stenotrophomonas maltophilia *in the presence of selenite. Therefore, the capability of strain CM100B to rapidly reduce soluble and toxic selenite (SeO_3_^2-^) to insoluble and unavailable Se^0 ^highlights it as a promising exploitable option for the setup of low-cost biological treatment unit for bioremediating selenium laden effluents. Although the red precipitate indicates the formation of elemental selenium (Se^0^), this fact does not exclude the possibility that there may be additional products, such as selenoamino acids and/or methylated selenides (gaseous products) formed during the selenite transformation process.

### Localization of reduced selenite (SeO_3_^2-^) in the bacterial cells

**Figure 4:**
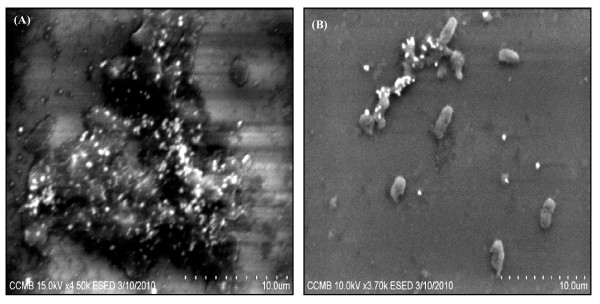
**(A) and (B) Environmental Scanning Electron Micrographs show the adherence of Selenium nanospheres to bacterial cell surface**.

**Figure 5:**
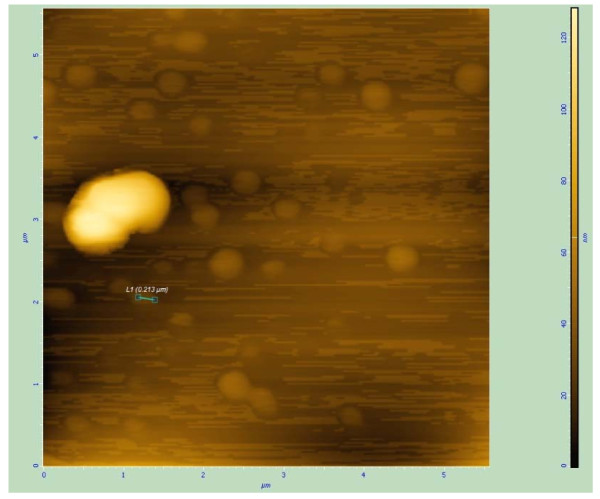
**Atomic Force Micrographs of Selenium nanospheres**. The bigger yellow structure is the single bacterial cell surround by Selenium nanospheres of size 150-200 nm dispersed in the culture medium.

**Figure 6:**
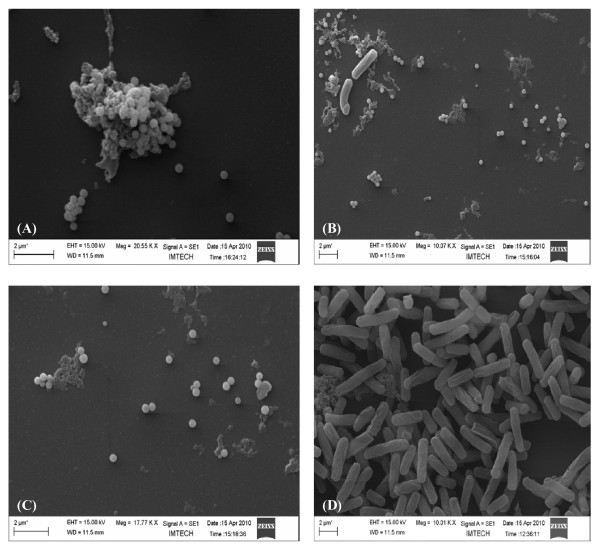
**Scanning Electron Micrographs of strain CM100B grown in the presence of 5 mM selenite**. **(A) **Selenium nanospheres adhering to the bacterial cells. **(B) **Bacterial cells surrounded by selenium nanospheres. **(C) **Free selenium nanospheres. **(D) **Control cells grown in TSB without addition of selenite.

Environmental Scanning Electron Microscopic (ESEM) study of bacterial culture obtained from the selenite-supplemented medium inoculated with strain CM100B revealed spherical structures associated with the cellular biomass (Fig. 4A and 4B). In Atomic Force Microscopic (AFM) imaging nanospheres of diameter ranging from 150-200 nm were observed in the extracellular medium (Fig. 5). Scanning electron microscopy (SEM) also showed spherical nanospheres of size ranging from 150-200 nm scattered around the cells as free deposits and also present as aggregates attached to bacterial cell mass (Fig. 6 A, B, C and 6D). Thus, the unique nature of this isolate is the potential to reduce the toxic selenite ions aerobically with concomitant generation of selenium nanospheres in the culture medium.

**Figure 7:**
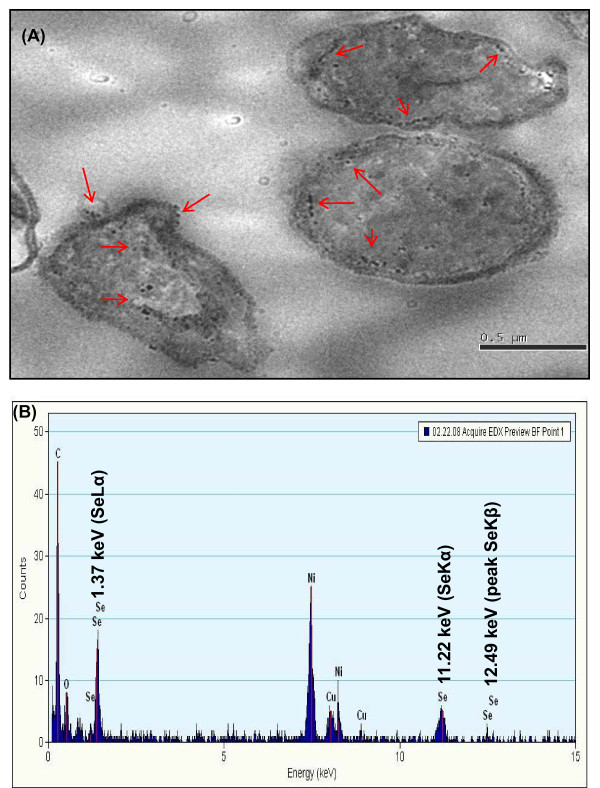
**TEM images of localization of selenium nanospheres (indicated by arrows)**. **(A) **Intracellular and surface localization of selenium nanospheres in strain CM100B under aerobic condition. **(B) **EDX spectrum of electron dense particles in the bacterial cells indicating the presence of selenium.

Transmission Electron Microscopy (TEM) of the cultures grown in the presence of selenite further confirmed the spherical intracellular and extracellular deposits of selenium nanospheres. (Fig. 7A).Studies have shown that active efflux of the metal is a frequently utilized strategy to produce tolerance by lowering the intracellular concentration to subtoxic levels [20]. However, our data showing intracellular nanometer-sized particles of elemental selenium (Se^0^) suggest that efflux pumps probably do not mediate the metalloid tolerance mechanism in strain CM100B since selenite tolerance is associated to an intracellular reduction of these oxyanions and then by their accumulation inside the cytoplasm or periplasmic space of the bacterial cell and subsequent exudation by the bacterial cell. The chemical microanalysis (TEM-EDX) of reddish colonies of strain CM100B grown in the presence of selenite revealed cytoplasmic and surface attached electron-dense Se^0 ^granules. In EDX analysis, the electron dense Se^0 ^particles produced specific selenium absorption peaks at 1.37 keV (peak SeLα), 11.22 keV (peak SeKα) and 12.49 keV (peak SeKβ) Fig. 7B. The uptake and transformations of selenite can be correlated to precipitation of selenium intra and extra-cellularly as observed by Lortie et al. (1992) [11]. The results are significantly similar to the observations by Kessi et al. [21] and Roux et al. [22], which indicated intense Electron Dispersion Spectroscopy (EDS) spectral peaks for SeKα at 11.22 KeV.

### Characterization of selenium nanoparticles (SNs) produced by strain CM100B

**Figure 8:**
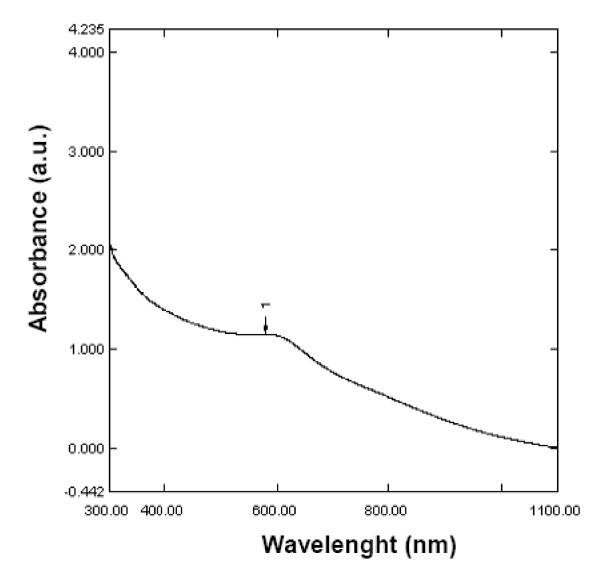
**Absorption spectra of selenium nanospheres isolated from *Bacillus cereus *strain CM100B**.

**Figure 9:**
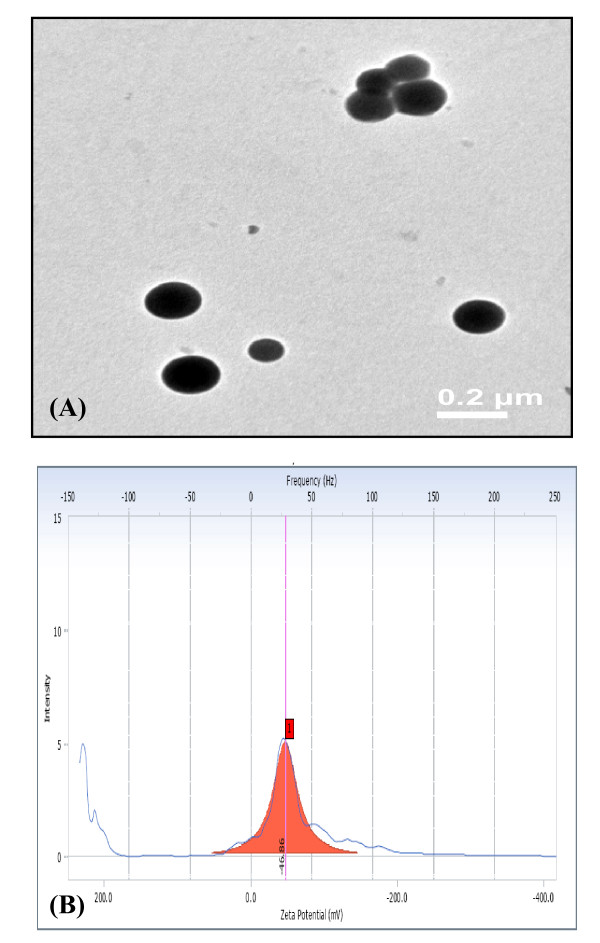
**(A) Transmission electron micrograph of Se nanospheres isolated from the culture broth**. **(B) **Zeta potential measurement of the selenium nanoparticles.

Elemental selenium (Se^0^) in the recent past has been envisaged to have immense medical (free radical scavenging, anti-cancer and anti-oxidative drug applications in medicine) and industrial (glass and optical lens coatings) applications. The growing importance of nano-selenium in diverse variety of applications has lured the researchers to study the biosynthesis of selenium nanoparticles. Therefore, further characterization of the selenium nanospheres produced by the strain CM100B was carried out. The UV-Visible absorption spectra of selenium nanospheres recovered from the culture broth gave characteristic peak at 590 nm which corresponds to particle size of 182.8 ± 33.2 nm [23] (Fig. 8). The size of SNs was further confirmed by TEM imaging which demonstrate that Se^0 ^particles possess an average diameter of 150-200 nm as depicted in Fig. 9A The zeta potential measurements indicate high negative charge (-46.86 mV) on the selenium nanoparticles (Fig. 9B)If all the particles in suspension have a large negative or positive zeta potential then they will tend to repel each other and there is little tendency for the particles to come together. However, if the particles have low zeta potential values then there is propensity of the particles to come together and form aggregates. The high negative charge on Se^0 ^particles is probably resulting in the high stability of the selenium nanospheres without forming aggregates and these particles do not transform to black amorphous form when kept for prolonged period of time of more than a month. The greater stability of allotropic form of Se^0 ^produced by bacteria or precipitated into cell-free medium obtained from the stationary phase culture implies that Se^0 ^is tightly bound to some substances produced by cells and is protected from further transformation to black form [21].

Pioneering study in characterizing the nano-hollow Se^0 ^spheres was carried out by Oremland et al. [14] who proposed that nanospheres are composed of interconnected three-dimensional nets of selenium in which both the chain and ring structural aspects are maintained, factors that resulted in the spherical shape of the nanospheres. Furthermore, the Se^0 ^particles precipitated by the three anaerobic bacteria (*Sulfurospirillum barnesii*, *Bacillus selenitireducens *and *Selenihalanaerobacter shriftii*) exhibited large variations in UV-visible and Raman spectral features, suggesting different species of Se-reducing bacteria produce Se^0 ^biominerals with different atomic structures. These structural variations were attributed to the diversity of enzymes that catalyze the reduction of selenium oxyanions. However, in another study under aerobic conditions, *Bacillus *sp. was reported to produce nano-structures showing hexagonal facet development and platy nanostructures. Se nanorods were also observed forming rosettes from Se nanospheres associated with the bacterial biomass [15]. Selenium biotransformation in the culture medium by *Enterobacter cloacae *cells resulted in formation of elemental Se^0 ^of size < 0.1 μm in diameter either free in the solution or protruding from the outer surface of the cells [24]. Kessi et al. [21] suggested that the presence of selenium particles on surface and in solution is an indication of vesicular mechanism to expel the bio-transformed selenium. Switzer-Blum et al. [25] examined the formation of small spheres of Se^0 ^on the cell surface of a gram-positive rod, *Bacillus selenireducens *strain MLS10, after respiratory growth on selenite. Reports of such formations are noted from *Stenotrophomonas maltophilia *[19], *Enterobacter cloacae *[24] and *Wollinella succinogenes *[26].

### Proposed mechanism of selenite (SeO_3_^2-^) detoxification and formation of selenium (Se^0^) nanospheres

The detoxification mechanism of selenite (SeO_3_^2-^) reduction in aerobic condition by microorganisms is not yet fully elucidated. More information is however available on the dissimilatory reduction pathways of selenite/selenate in anoxic environments. Microbial transformations of selenium oxyions (selenite/selenate) to insoluble forms such as elemental Se^0 ^may not be the only end product in transformation process as assimilation of organic forms such as selenoamino acids and reduction and methylation of selenium oxyions which yields volatile products, primarily dimethyl selenide has also been observed in some bacterial species [27,28]. Other organometallic forms of selenium like dimethylselenide and dimethyldiselenide are produced by *Rhodospirillum rubrum *and *Rhodocyclus tenuis *while growing phototrophically in the presence of selenate [29]. *Pseudomonas fluorescens *K27- a gram negative denitrifying facultative anaerobe isolated from the Kesterson Reservior, California has been reported to detoxify metalloids: Se, Te, Sb which are reduced to the elemental form and further to some extent to -2 oxidation state along with biomethylation. The presence of volatile compounds of Se: dimethyl selenide (DMSe, CH_3_SeCH_3_), dimethyl diselenide (DMDSe, CH_3_SeSeCH_3_), dimethyl selenenyl sulfide (DMSeS, CH_3_SeSCH_3_) have been reported in the headspace of the cultures amended with soluble selenium salts [30].

**Figure 10:**
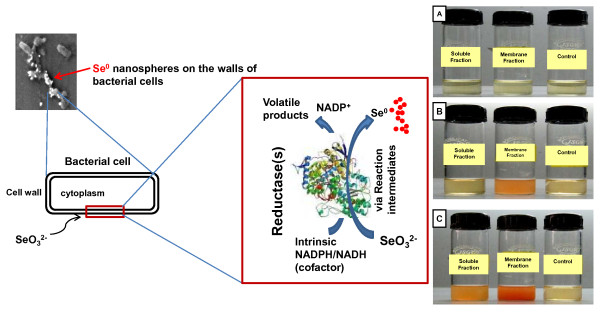
**Schematic representation of proposed mechanism of biogenesis of Selenium (Se^0^) nanospheres**. **(A) **Selenite reduction at 0 h. **(B) **Formation of red elemental selenium in membrane fraction after 3-4 h of incubation. **(C) **Prolonged incubation of 12 h resulted in formation of red elemental selenium in soluble fraction also.

Majority of metal transformations in anaerobic and aerobic environments are the result of the direct enzymatic activity of bacteria [31]. The bacterial reduction of selenate (SeO_4_^2-^) to selenite (SeO_3_^2-^) is known to occur through dissimilatory pathways [12,24,32], the reduction of selenite (SeO_3_^2-^) to Se^0^, which is a common feature of many diverse microorganisms, is also not well understood. In the present study selenite reduction activity was observed mainly in the membrane fraction when it was incubated in the presence of selenite as a substrate [Fig. 10B]. However, there was nominal activity in the soluble fraction after prolonged period (12 h) of incubation at 37°C [Fig. 10C]. This may be attributed to some non-specific reduction of selenite or by the diffusible nature of the membrane associated reductase protein(s) which resulted in selenite reducing activity in the soluble fraction. Previously, it has been reported that SeO_3_^2- ^reduction may be catalysed by a periplasmic nitrite reductase [13,33], hydrogenase I [34] or through non-enzymatic reactions [26]. Lortie et al. [11] reported that the aerobic reduction of selenate (SeO_4_^2-^) and selenite (SeO_3_^2-^) to Se^0 ^by a *Pseudomonas stutzeri *isolate functioned as a detoxification mechanism as there was no evidence of dissimilatory Se reduction by this microbe. Recent studies have indicated that NADPH/NADH dependent selenate reductase enzymes bring about the reduction of selenium (selenite/selenate) oxyions [35,36]. In the medium where tellurite or selenate was absent no growth was observed but growth occurred in the presence of tellurite or selenate indicating that these reductases are probably respiratory enzymes. The reduction seems to be initiated by electron transfer from the NADPH/NADH by NADPH/NADH-dependent reductase as electron carrier. In some bacterial species selenite reduction may serve functions of detoxification and maintenance of redox component of the electron transport system by cytoplasmic reductase enzymes [10,37,38]. Evidence of selenite/selenate reduction via membrane-associated reductase(s) followed by rapid expulsion of selenium particles was reported by Losi and Frankenberger [10]. On the basis of experimental observations and the available literature the possible mechanism of formation of selenium nanospheres by the strain CM100B is proposed in Fig. 10. The toxic selenite anions are probably being reduced by membrane associated reductase(s). The transformation process is carried out via reaction intermediates which are still uncharacterized. The end product of the transformation process results in volatile methylated forms of selenium and formation of selenium nanostructures which are being expelled out in the culture broth by the bacterial cells. However, the exact mechanism of the reduction of selenite oxyions is yet to be deciphered completely. Clearly, additional work is needed to elucidate the enzymology involved in the reduction processes of selenium oxyions in aerobic conditions.

## Conclusions

Several potential advantages revolve around the microbe's ability to grow in aerobic conditions which include rapid ability to generate more number of bacterial cells within a short time period and less stringent culture conditions. The aerobically produced nanoparticles by the microbe *Bacillus cereus *(strain CM100B) have been characterized in this study. Biosynthesis of amorphous Se^0 ^nanospheres under aerobic conditions offers advantages over chemical processes, in which amorphous Se^0 ^is produced under environmentally harmful conditions. The strain tolerates high levels of selenium oxyions and generates extracellular nanospheres of selenium (~ 150-200 nm in diameter) which can be easily separated from the bacterial biomass by a simple centrifugation step without any post preparative treatment. The amorphous selenium nanospheres formed during the aerobic detoxtification of selenite by the strain CM100B were observed to be highly stable due to the presence of high negative charge. This green route of biosynthesis of selenium nanospheres is a simple, economically viable and an eco-friendly process resulting in nearly monodispersed highly stable selenium nanospheres. Further studies would determine if the diverse properties of the biologically based selenium nanospheres are comparable to chemically synthesized selenium nanoparticles and whether biologically synthesized selenium nanoparticles have practical applications in the field of nanotechnology and biotechnology.

## Competing interests

The authors declare that they have no competing interests.

## Authors' contributions

SD planned and performed the experiments. SSC supervised the work. SD and SSC wrote the final version of the manuscript. The authors approved the final version of the manuscript.
